# What Is Dental Literature?

**Published:** 1919-08

**Authors:** Garrett Newkirk

**Affiliations:** Pasadena, Cal.


### WHAT IS DENTAL LITERATURE?

BY GARRETT NEWKIRK, M.D., PASADENA, CAL.

It must be admitted that all writing relating strictly to the teeth comes under the head of dental literature.

Their anatomy, physiology, histology, hygienic relations; everything pertaining to their conservation; all forms of treatment and restoration, with endless details relating to materials and their manipulation—all is included. The scope of the subject can hardly be imagined even by those who have given it years of study. Yet, by the evolution of practice, by natural specialization, dentistry involves more. It has taken from the general practitioner the entire oral field. It is expected, and even demanded, of the dentist to-day that he alone shall be the chief authority on the pathology, medication and surgery of the mouth. He joins hands with the rhinologist, the oculist, the otologist and the general practitioner. Moreover, dentistry extends to relations of art and esthetics; to voice and expression.

In its restorative measures are involved studies of metallurgy, mechanism, intricate and delicate, and the genius of invention.

So much for the special, acknowledged field of dental literature. But having gone so far, we can not avoid going further. Dentistry shares in common with general medicine all there is of the basal sciences, anatomy—human and comparative—physiology, chemistry, physics and hygiene. Its literature reaches forth to every practical relation with these subjects. Is the field broad enough, think you?

Dental literature, like other forms of literature, consists of two classes: First, that of current periodicals, consisting of papers, discussions, records, descriptions, editorials. Second, matter supposedly valuable is printed and bound into books for preservation and convenient reference. These books assume to be in some sense authorities on the subjects treated. With formal books are included volumes of the transactions of various societies.

Books contribute little matter to the periodicals; journals on the contrary contribute largely to the making of books. Matter intended for a book is sometimes published serially in a magazine for profit or advertisement. But whatever the preliminaries, the book should possess certain characteristics. It first should meet a definite need of the profession as a whole or some branch thereof; temporarily at least if not permanently. If only the former, it will soon be forgotten, and will exist later only as a curiosity of the literary museum. Most books, good at the time of their publication, are likely to be superseded. They must possess very high value indeed to hold place long. The last word on any subject is hard for any one to print. A Gray in anatomy, a Gray in botany, a Coues in ornithology, a Black in dentistry—such are figures for the centuries to measure from. A book should have originality. The personal equation counts not a little. Something of character goes with it. Consciously or unconsciously one reads between the lines qualities of mind and heart. This involves sincerity, singleness of purpose and helpfulness. A book should present some new truth, or old truth in better form, for instruction and practical use.

Verbiage should be avoided. The author should labor diligently and take no end of pains to have his expression fit; to find the right word and to use the least number that will make his meaning clear. His expressions should illuminate and not confuse the mind of the reader. He should strive for clear, simple and never long or involved sentences. Even the Apostle Paul could have improved his style greatly in this respect. And sometimes a little humor, if not dragged in by the heels, may help a dry subject.

Any book worthy of publication should have careful editing, at least reading and suggestion, by some other than the author. No one can write so perfectly that another far less capable perhaps may not discover an error or possibility of improvement. Not one word of a book should go unchallenged by the merciless eye of a careful proofreader or critic.

Jenner worked twenty-three years on his vaccination treatise of seventy pages, giving details of twenty-three successful cases.

Bryant wrote Thanatopsis, it is said, a hundred times. Goldsmith thought four lines an unusual day's work, and had "The Deserted Village" in hand seven years. Seldom is good writing spontaneous, or without great labor. Most authors do not take pains—in the literal, true meaning of the term—and one has said, "If you do not take pains, pains will take you."

Any book containing matter worthy of reference should be carefully indexed, as to all facts, subjects, individuals spoken of and authorities quoted. Nothing is more annoying than to want something that you know is in a book yet you can not find it promptly, because not indexed.

Whatever illustrations are necessary to the understanding of the text should be placed in proper relation. Care should be exercised *not* to over-illustrate, so that the reader is not a reader, but thinks he has it all in pictures while he turns the leaves like the reel of a "movie."

I well remember the first description I read of Taggart's inlay process. There were no pictures but of words, so well written by himself that nothing more was needed. I could make my own pictures as I read, and I exclaimed with exultation, *"That is what we've been looking for!"* How much better this than so-called illustrations that we often see of impossibe anatomy, or appliances that look as if made by a cross-roads blacksmith.

I am inclined to emphasize my positives with a few negatives.

(1) "A writer should *not* permit himself to write hurriedly, without concentration, revision and correction." He should never rush a book into print, until it is as nearly perfect as he could hope to make it.

(2) His sole purpose should be to state the truth, and get it *to* his readers; *not* in large part to gratify personal ambition.

(3) It should *not* be written with the idea of promulgating some pet theory of his own, while important facts or views of others are withheld or twisted.

I well remember the early periods of most leading dental journals. In the files of the *Dental Cosmos*, beginning with its mother, the *Dental News Letter*, is to be found the largest part of dental history for seventy years. I have tried to encourage dental students to read these old journals, in order to learn something of the men who built the profession. They were "giants in those days"—those of whom our young men know but little. Through the pages of these journals one may trace the evolution of dental thought in its entirety. I insist on giving this advice to students and the young practitioners, even if they pay no attention to it.

The number of dental journals now printed in the United States is quite beyond my reckoning. Nearly every one is given at a price of one dollar or less. Each is worth what it costs or a good deal more to the subscriber who reads. I marvel not at what they lack, but rather at their wealth of content.

Almost every leading dental journal is backed by a commercial interest. Otherwise none could exist with a subscription price of one dollar.

The commercial and manufacturing house pays the editor and the printer, in consideration of the advertising pages and the reader gets his journal for practically nothing. Of course, in the end, "Jones, he pays the freight," as he does the tariff on his tea, unconsciously. But as a rule the house is pretty fair; the editor is honest and not conscious of servitude.

It is needless, perhaps, to remind ourselves that most of the matter of our periodicals consists of papers read before dental societies, with their discussions. The organization of many new societies has increased correspondingly the number of such papers. In Illinois, for instance, there are now twenty-seven local organizations instead of five or six a few years ago. So there are being written, read and discussed five times as many papers, perhaps.

Are these all worth publication in our periodicals? Certainly not.

Is it worth while that they should be written and delivered? Certainly it is. It is necessary to the interest and to the very existence of each society that papers be written by its various members. These may not be necessarily of high quality or literary excellence. An indifferent paper may occasion a valuable discussion, or its tone may promote good will and harmony. It is the *spirit* of the writer that counts most. Moreover, it is worth while for the awakening, encouragement and development of the writer himself. He is the better for his own effort. He has made an advanced step. By the writing of his first paper, with doubt and timidity perhaps, many a one has discovered himself, and opened up a new career of usefulness and happiness.

But in the worst written paper there is likely to be something of value; a practical suggestion not only for the members of the local society, but thousands more if they had it presented to them in print.

It seems to me that we ought to have some system for saving these fragments.

It could hardly be expected that the local society should always have among its members a first-class editor. If so fortunate, of course, his services would be invaluable. But suppose the state association were to appoint an editor of unquestioned ability, to whom all papers unsolicited by publishers should be submitted—always typewritten; suppose he were to go over these one by one through the year, making brief notes or quotations; and suppose that at the annual meeting he should present a summary of what he had discovered; would it not be worth while? There ought to be some one in every state who would love to do this work, and in the interests of conservation the profession could even afford to pay him a small sum for his service.

In passing we may say that an abbreviation of many papers now published in whole would diminish cost of printing and be a refreshment to the reader.

What has been said with reference to the making of books applies to the writing of articles for societies or journals. The editor is often sorely tried, or fairly appalled by the prolixity, useless repetition and the careless slinging together of words in the manuscripts submitted for his use. And he publishes stuff, perforce, that he knows in justice to the reader and the author and himself should be entirely rewritten.

What encouragement is there for the publisher of dental books? Very little, I fancy. I am not very much in the confidence of those gentlemen, but it is my impression that they have grown wary of the risk. I am sure that as a rule such books have not paid. Personally I know of but two, one on operative dentistry, another on orthodontia, that have been fairly remunerative to authors or publishers.

Why? We may as well face the answer squarely, unflattering though it may be. Dentists as a rule *do not read,* and they *will not buy,* books. They are not students of the literature of their own profession nor of the wider literature of general medicine and surgery. As a rule they are not very familiar with any sort of literature beyond the daily newspaper or some dental magazine which contains as they think the greatest number of paragraph articles—that they call "practical." They have no use for any reading which does not seem to have an immediate relation to the dollar they are after. How many, think you, out of a hundred have ever read studiously one standard dental or medical book?

I have not, I think, the disposition of a pessimist or a "knocker." On the whole we may be making progress, but I am not disposed to any Fourth of July celebration in these conventions—with fire-crackers and rockets or spread-eagle oratory about our "glorious history" and wonderful advance.

I know of one book that ought to be in the library of every dentist, for reading and reference; an authoritative book. It fulfills the conditions of a good book in matter and literary style. I imagine no publisher was willing to undertake it, for it was privately printed at the author's expense. I presented this book to the attention of fifty students, so called. They admired it and admitted its value, but not one would invest therein two dollars. Neither would the practicing dentist buy it.

Dr. Arthur D. Black, a few months ago, read a paper before the American Institute of Dental Teachers at Ann Arbor, entitled, "Suggestions for Making the Dental Student a Better Student Dentist."

It was printed in June number of the *Dental Review*, 1915—how many present heard or have read the article? It is worth while perhaps to quote a few sentences from this remarkable paper:

"We must realize that the dentist graduated without having been taught to study will not be equipped to keep pace with the progress of events."

"We must develop a new type of dentist." "He must be essentially and especially a man who *thinks*."

His criticism of our present system of dental education is that students listen for hours to talk, talk, talk, with more or less of occular demonstration, but they are not taught to read, to study, to reflect, to master problems by their own mental processes. They read only to cram for an examination. Dr. Black proposes the substitution in large measure of a recitation system, wherein the student shall acquire information far more than he does now by books. And the most important thing about this is that the student forms a habit for his whole professional life. He says: "If a man is trained during three or four years to get his knowledge from books, to spend his evenings with them, to have them for his constant companions instead of having all his information poured in from a rostrum, or crammed in under examination pressure, he will have learned to depend on books, and will be more likely to use them in the future.''

I will add to this statement of Dr. Black what naturally follows: that if the student practitioner reads books, he will have a desire to own them; he will buy a few; the publisher could count on publication without loss; and the writer of a really valuable book would secure a royalty that would pay possibly half what a plumber gets per hour for his time.

Whatever the outcome may be of Dr. Black's plan, any reader of his paper will have stimulus for thought.

A man's vocation should lead in his reading as it does in his life. Why should not medico-dental literature, book or periodical, have first claim, over and beyond the daily newspaper, the story magazine or the always ephemeral ''best seller''? Do you remember when every third person you met would ask, ''Have you read Trilby''?

What relative interest have you or I as to who won the impossible female that a mercantile writer created and named ''Barbara West''?

We shall never be gloriously free until, when we are asked by Tom, Dick or Harry, ''Have you read?''—this or that book of fiction—we are able to respond with exultation. ''*No!* thank the Lord, I have not.''

To read literature of importance with benefit, surely one must be able to think as he reads. This involves aloneness, quiet, *time.* To obtain this seems to be increasingly difficult for most of us. We are living in the age of greatest distractions. Recent discoveries have multiplied complications that get their grip on everybody.

A wise friend said to me not long since: ''Talk about the simple life; there isn't any such thing. One is called on every minute to do something, answer somebody, and to decide questions immediately that will not wait. The decision may be wrong, but no matter, he must make it.''

My friend was not far from the truth. He has since yielded up his own life to what he called his ''*besetments.*''

Within one or two decades the world seems to have gone *power mad* and *speed crazy*. Nations, like men, and with men have grasped these new forces of science, and applied them, not to conservation, but destruction. They are being made the instruments of hate and wholesale murder on a scale never dreamed of hitherto.

What we call efficiency, and worship as such, has been organized to make hell on earth. It has made whole nations apparently insane, without judgment or right feeling. The civilized world, so called, is dazzled with new science till the sun of righteousness is well-nigh eclipsed.

What chance has real literature that requires the spirit of repose and reflection? What has become of the fireside, the quiet home circle, the reader and the listener?

From the time our children enter the grammar school at six till they leave high school at eighteen they are crowded with ever-increasing multiplicities. Essential unities are likely to be lost out of sight. So-called students have no time for going deep into anything. Outside of school they demand only amusement, excitement, thrills.

Why should they *read* natural history, when they can see the ''real thing'' in the ''movies''? Who among them knows the meaning of ''the classic''? A high school student will ask if Aesop is living, or a Dutchman? (The handsomest, best-groomed young dentist I ever knew, said that he had often heard of Dickens, and he meant some day to read the book.)

What use is the old road? Take a short cut through the brush.

Why read a standard book with stillness, when one has the ''Compend'' all in short paragraphs; or the quiz-class, with lively company? Do nothing alone. Why read history or political economy? Go with ten thousand others and listen to Bryan. He will tell you all that is worth knowing in an hour.

What use have we for the old past anyway? This is a *new* age with law unto itself. Everything has changed—except the universe—and human nature.—*Pacific Dental Gazette.*

